# Association between Diabetes and Risk of Aortic Dissection: A Case-Control Study in a Chinese Population

**DOI:** 10.1371/journal.pone.0142697

**Published:** 2015-11-12

**Authors:** Xingwei He, Xintian Liu, Wanjun Liu, Bei Wang, Yujian Liu, Zhuxi Li, Tao Wang, Rong Tan, Bo Gao, Hesong Zeng

**Affiliations:** 1 Department of Cardiology, Tongji Hospital, Tongji Medical College, Huazhong University of Science and Technology, Wuhan, China; 2 Department of Cardiology, Suizhou Affiliated Hospital of Hubei Medical College, Suizhou, China; Katholieke Universiteit Leuven, BELGIUM

## Abstract

**Background:**

It is well-recognized that diabetes represents a powerful independent risk factor for cardiovascular diseases. However, very few studies have investigated the relationship between diabetes and risk of aortic dissection (AD).

**Aim:**

The aim of this case-control study was to evaluate the association between diabetes and risk of AD in Chinese population.

**Methods:**

A hospital-based case-control study, consisting of 2160 AD patients and 4320 controls, was conducted in a Chinese population. Demographic, clinical characteristics and risk factors were collected. Diabetes rate of patients with overall AD, Stanford type A AD and type B AD group was compared with that of corresponding matched control groups. Logistic regression analysis was used to estimate the odds ratios (OR) and 95% confidence intervals (95% CI) for relationship between diabetes and AD risk.

**Results:**

The prevalence of diabetes was lower in AD cases than that of control subjects, whether it is the overall AD, type A AD or type B AD group (4.7% vs. 10.0%, 2.9% vs. 8.8%, 5.9% vs. 10.9%, all P<0.001). Furthermore, in multivariate model, diabetes was found to be associated with lower AD risk, which not only applies to the overall AD (OR = 0.2, 95%CI: 0.15–0.26), but also type A AD (OR = 0.12, 95% CI: 0.07–0.20) and type B AD (OR = 0.25, 95%CI: 0.18–0.33).

**Conclusions:**

We observed the paradoxical inverse relationship between DM and risk of AD in the Chinese population. These results suggest diabetes may play a protective role in the development of AD. However, further studies are needed to enrich related evidence, especially with regard to underlying mechanisms for these trends.

## Introduction

Aortic dissection (AD) is a potentially critical break in the lining of the main arterial outflow from the heart [1]. As a relatively uncommon yet catastrophic disease, it affects 5 to 30 per 1 million people annually, amounting to nearly 10,000 cases in the United States [2–4]. According to the literature, 20% of the patients with AD die before reaching hospital and 30% die during hospital admission [5]. Although AD is frequently fatal, the precise etiology remains unclear and many diseases are considered to be associated with it. Data from International Registration Aortic Dissection (IRAD) revealed that hypertension and atherosclerosis were the most common predisposing factors for AD, followed by old age, and previous cardiovascular surgery, Marfan syndrome, and iatrogenic causes [1, 2, 6].

Diabetes is a high risk factor for the development of cardiovascular diseases (CVD)and atherosclerosis [7, 8]. Numerous clinical studies have shown a direct correlation between the level of hyperglycemia and CVD morbidity and mortality. Besides, it has also been shown that there exists a significant association between the degree of hyperglycaemia and increased risk of microvascular complications, macrovascular mortality, and all-cause mortality in patients with diabetes [9–12]. Based on the above analysis, diabetes seemed to be considered as a risk factor for AD.

However, opposed to the assumption, a few recent studies indicated that diabetes has a protective effect against aortic diseases, including AD [13–15]. The result is, to a certain extent, beyond many researchers’ expectation and has brought about significant impacts. Therefore, it can be noted that those studies reached inconsistent conclusions on the relationship between diabetes and AD risk. In this study the hypothesis is that diabetes serves a positive role in the development of AD risk and we performed a retrospective case-control study with Chinese population as subjects.

## Materials and Methods

### Ethics Statement

The study was approved by the Ethics Committee of the Tongji hospital of Huazhong University of Science and Technology. All aspects of the study comply with the Declaration of Helsinki. Ethics Committee of Tongji hospital of Huazhong University of Science and Technology specially approved that not informed consent was required because data were going to be analyzed anonymously.

### Study Population

This project was designed as a hospital-based retrospective 1:2 case—control study. Between 1 January 2003 and 1 December 2013, a total of 2160 consecutive AD patients at Tongji Hospital (Wuhan, China) were enrolled in the study. Cases were diagnosed by imaging, surgical visualization, or autopsy. Patients with traumatic aortic dissection and iatrogenic aortic dissection were excluded. According to the Stanford classification system [16], the cases were divided into two categories: those involving the ascending aorta (type A AD, n = 861) and those not involving the ascending aorta (type B AD, n = 1299).

In addition to describing case subject’s characteristics, a case—control approach was performed. 4,320 controls (3,352 males and 968 females) were selected at random by frequency matching age (plus or minus 1 year) and gender from 22,430 people (12,084 males and 10,346 females), who had received a health examination in Tongji hospital in 2011. All patients with previous history of aortic dissection, aneurysm and active inflammatory disease were excluded to remove elements of confounding bias. The ratio of cases to controls was 1:2. The controls were divided in groups according to the different case groups. The corresponding controls for overall AD group were controls group (n = 4,320), for type A AD group were control A group (n = 1,722) and for type B AD group were control B group (n = 2,598). Information on demographic and other variables, such as diabetes mellitus (type 2), hypertension, hyperlipidemia, history of smoking, Marfan syndrome, bicuspid aortic valve, COPD, etc, were abstracted from hospital charts. The prevalence of diabetes in each AD group was compared with that of the corresponding control groups, respectively. Finally, the odds ratios (OR) and 95% confidence intervals (95% CI) for relationship between diabetes and risk of AD in each case/control group were calculated.

#### Definitions used in the study

History of smoking was assumed if the patient had smoked within the last 10 years. Hypertension was defined as blood pressure ≥140/90 mmHg or treatment for hypertension before admission; hyperlipidemia was defined as cholesterol level >220 mg/dl or treatment for hyperlipidemia; According to the WHO criteria [17], diabetes was defined either by fasting plasma glucose levels ≥126 mg/dl, 2-h post-load glucose levels ≥200 mg/dl after a 75goral glucose tolerance test, or by physician diagnosis; Marfan syndrome was diagnosed according to the revised Ghent criteria [18]. Patients were evaluated according to the ACC/AHA guidelines in order to assess the presence of coronary artery disease (CAD).

### Statistical analysis

Continuous variables are presented as mean ± SD, and categorical variables are presented as frequencies. All comparisons between two groups of continuous variables were made using a two-sample t-test or nonparametric test; the Chi-square test was employed when comparing categorical variables. To study the association between diabetes and AD risk, logistic regression method was used for the calculation of odds ratio (OR) as well as its 95% confident interval (95% CI). Adjusted ORs were computed using multivariate logistic regression with adjustment for history of smoking, hyperlipidemia, hypertension, coronary heart disease, peripheral vascular disease, drug abuse, Marfan syndrome, bicuspid aortic valve and COPD. P<0.05 was taken to indicate statistical significance. The statistical analyses above were performed with the SPSS Software version 16.0 (SPSS, Chicago, IL, USA) and were based on two-tailed probability.

## Results

**Fig 1:**
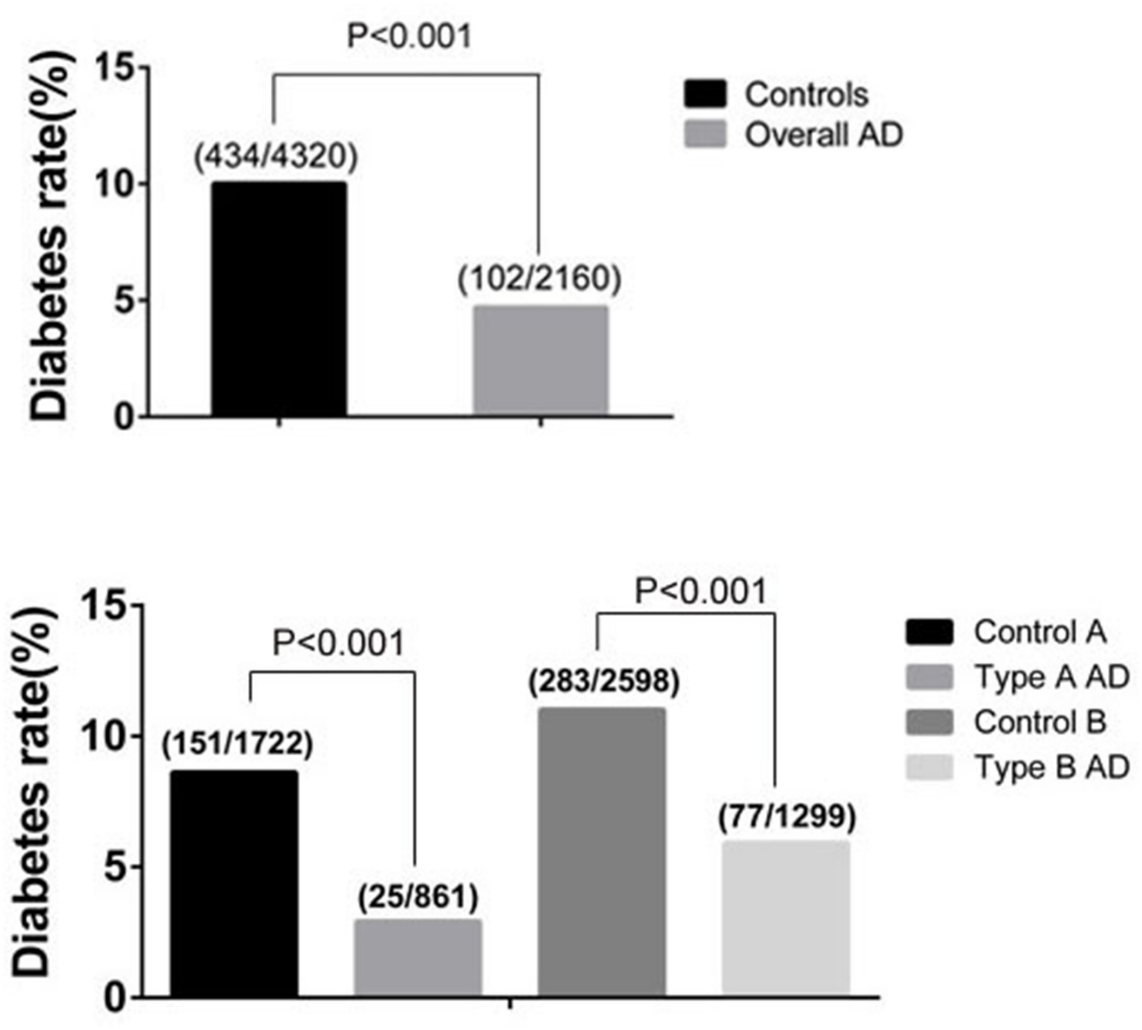
Differences of diabetes rate between AD patients and control subjects.

The baseline characteristics of the case/control samples are shown in Table 1. The mean ages of case and control group were 53.6±12.0 and 53.6±12.2 years respectively with males accounting for 77.6% of each group. No significant difference in age and gender was detected between each case/control group, which shows that the frequency matching was adequate. In terms of comorbidities, history of smoking, hypertension, hyperlipidemia, Marfan syndrome, bicuspid aortic valve, coronary heart disease, drug abuse and COPD were observed more frequently in the AD patients. However, the control subjects presented with diabetes more frequently than the AD patients (10% vs 4.7%, p<0.001) (Fig 1). According to the national survey in 2010 [19], the incidence of diabetes in Chinese adults aged over 18 years old was 11.6%. The diabetes rate in control group was close to that of general Chinese adults.

**Table 1:** Characteristics of patients with AD and of matched controls.

Characteristics	Overall AD			Type A AD			Type B AD		
	Controls	AD	P value	Control A	Type A AD	P value	Control B	Type B AD	P value
**N**	4,320	2,160		1,722	861		2,598	1,299	
**Age, y**	53.6±12.2	53.6±12.0	0.850	52.4±12.5	52.3±12.6	0.833	54.3±12.0	54.5±11.4	0.660
**Male, %**	77.6	77.6	1.000	73.4	73.4	1.000	80.4	80.4	1.000
**BMI, kg/m** ^**2**^	24.9±3.4	25.0±3.5	0.052	24.9±3.5	25.1±3.5	0.151	24.9±3.3	25.0±3.4	0.180
**History of smoking, %**	30.4	56.3	<0.001	29.2	51.2	<0.001	31.3	59.7	<0.001
**Hyperlipidemia, %**	5.1	11.2	<0.001	5.1	8.0	0.004	5.2	13.2	<0.001
**Hypertension, %**	18.9	72.3	<0.001	17.1	65.5	<0.001	20.1	76.8	<0.001
**CHD, %**	3.6	4.7	0.035	2.9	4.8	0.018	4.0	4.6	0.397
**PVD, %**	3.1	4.0	0.057	2.8	4.2	0.077	3.2	3.8	0.351
**Drug abuse, %**	0.1	0.5	0.006	0.1	0.2	0.259	0.2	0.7	0.014
**Marfansyndrome, %**	0.1	1.7	<0.001	0.2	2.2	<0.001	0.1	1.4	<0.001
**Bicuspid aortic valve, %**	0.1	0.7	<0.001	0.1	1.2	<0.001	0.1	0.4	0.127
**COPD, %**	9.3	16.0	<0.001	8.7	15.4	<0.001	9.7	16.4	<0.001

Data are presented as the means ± SD or %; AD, aortic dissection; BMI, body mass index; CHD, coronary heart disease; PVD, peripheral vascular disease; COPD, chronic obstructive pulmonary disease.

We further divided cases into two subgroups, Stanford type A and type B. The corresponding controls for type A AD group were control A group and for type B AD group were control B group. Similarly, the prevalence of diabetes in Stanford type A AD and type B AD group were lower than that of control A and control B group (8.8% vs. 2.9% and 10.9% vs. 5.9%, all P<0.001).

Table 2 presents the difference between diabetes and non-diabetes groups in the study population. For both AD patients and control subjects, patient with diabetes were older in age than those without; Hypertension and Peripheral vascular disease (PVD) were higher in diabetes group (P<0.001); Furthermore, in the control subjects, hyperlipidemia and CHD were higher in diabetes group than in those without (p <0.001). However, there was no significant difference with regard to other demographic variables.

**Table 2:** Comparison in characteristics between diabetes and non-diabetes groups in the study population.

Characteristics	Patients with AD			Control subjects		
	Diabetes	Non-diabetes	P value	Diabetes	Non-diabetes	P value
**N**	102	2058		434	3886	
**Age, y**	59.4±12.3	53.3±11.9	<0.001	58.23±11.9	53.05±12.17	<0.001
**Male, %**	70.6	77.9	0.089	81.1	77.0	0.069
**BMI, kg/m** ^**2**^	25.0±3.3	25.0±3.5	0.867	25.0±2.5	24.9±3.0	0.358
**History of smoking, %**	56.9	56.3	0.919	29.5	30.5	0.700
**Hyperlipidemia, %**	14.7	11.0	0.258	11.5	4.4	<0.001
**Hypertension, %**	94.1	71.2	<0.001	46.5	15.8	<0.001
**CHD, %**	8.8	4.5	0.052	11.1	2.7	<0.001
**PVD, %**	16.7	3.4	<0.001	5.8	2.8	0.002
**Drug abuse, %**	0	0.5	1.000	0	0.1	1.000
**Marfansyndrome, %**	3.9	1.6	0.094	0	0.1	1.000
**Bicuspidvalve, %**	0	0.8	1.000	0	0.1	1.000
**COPD, %**	15.7	16.0	1.000	10.8	9.1	0.257

Data are presented as the means ± SD or %. AD, aortic dissection; BMI, body mass index; CHD, coronary heart disease; PVD, peripheral vascular disease; COPD, chronic obstructive pulmonary disease.

In multivariate logistic regression analysis, after adjustment for hypertension, history of smoking, Marfan syndrome, hyperlipidemia, bicuspid aortic valve, drug abuse, CHD, PVD and COPD, DM was related to the reduced risk of AD (adjusted OR = 0.20, 95%CI: 0.15–0.26). Furthermore, the inverse association also remained significant in the Stanford type A/case A group (OR = 0.12, 95%CI: 0.07–0.20) and type B/case B group (OR = 0.25, 95%: 0.18–0.33). Other factors that were independently associated with risk of AD are shown in Table 3.

**Table 3:** Logistic regression analysis for risk factors of overall AD, type A AD and type B AD.

	Overall AD (n = 2,160)			Type A AD (n = 861)			Type B AD (n = 1,299)		
	AOR	95% CI	P value	AOR	95% CI	P value	AOR	95% CI	P value
**Diabetes**	0.20	(0.15–0.26)	<0.001	0.12	(0.07–0.20)	<0.001	0.25	(0.18–0.33)	<0.001
**History of smoking**	2.80	(2.44–3.20)	<0.001	2.50	(2.02–3.11)	<0.001	3.09	(2.59–3.70)	<0.001
**Hyperlipidemia**	2.14	(1.69–2.71)	<0.001	1.48	(0.99–2.19)	0.055	2.71	(2.01–3.66)	<0.001
**Hypertension**	13.10	(11.47–14.96)	<0.001	10.99	(8.94–13.50)	<0.001	15.46	(12.95–18.45)	<0.001
**CHD**	0.80	(0.59–1.10)	0.165	1.16	(0.70–1.94)	0.568	0.63	(0.42–0.94)	0.023
**PVD**	1.05	(0.74–1.48)	0.792	1.63	(0.96–2.77)	0.071	0.80	(0.51–1.24)	0.314
**Drug abuse**	3.39	(1.00–11.56)	0.051	3.26	(0.24–44.51)	0.376	3.71	(0.91–15.20)	0.068
**Marfan syndrome**	37. 28	(12.78–108.74)	<0.001	23.30	(6.61–82.16)	<0.001	74.29	(9.30–593.65)	<0.001
**Bicuspid aortic valve**	5.07	(1.59–16.19)	0.006	8.92	(1.71–46.56)	0.009	1.97	(0.33–11.67)	0.454
**COPD**	0.90	(0.74–1.10)	0.290	0.86	(0.62–1.19)	0.352	0.94	(0.73–1.21)	0.641

AD, aortic dissection; CHD, coronary heart disease; PVD, peripheral vascular disease; COPD, chronic obstructive pulmonary disease. AOR, adjusted odds ratio; CI, confidence interval.

Finally, we compared the incidence of in-hospital death between diabetes and non-diabetes in AD patients. As shown in Table 4, there was no significant difference among the groups with respect to the incidence of in-hospital death. 223 of 2058 (10.8%) non-diabetic patient died due to AD, whereas 14 of 102 diabetic patients (13.7%) died (P>0.05).

**Table 4:** In-hospital AD death of diabetes group and non-diabetes group.

	Non-diabetes	Diabetes	P value
**Mortality in overall AD**	223 of 2058	14 of 102	0.333
**Mortality in type A AD**	167 of 836	8 of 25	0.203
**Mortality in type B AD**	56 of 1222	6 of 77	0.261

AD, aortic dissection.

## Discussion

Diabetes has been known to be a powerful risk factor for associated cardiovascular diseases (CVD) and the development of atherosclerosis. Patients with type 2 diabetes have a 2- to 4- fold higher risk of CVD death compared with patients without [11]. Furthermore, to our knowledge, diabetes is also highly related with hypertension. In our study, for all patients with AD, diabetes group were at higher risk of developing hypertension and PVD, and they are older in age compared with non-diabetics group. It has been reported that history of hypertension, old age, and atherosclerosis are the most common risk factors for AD [1]. Hence, diabetes seems to be considered as a risk factor for AD. But this is not the case.

Previous studies have shown that for patients with high risk of atherosclerosis, forbidding smoking and controlling the blood pressure and blood lipid in normal level can significantly reduce cardiovascular morbidity and mortality. However, with the hemoglobin A1c (HbA1c) controlled at normal level for patients with diabetes and high risk of atherosclerosis, the incidence of micro-vascular disease may be reduced, but a significant increase in mortality of macro-vascular can be brought about [20, 21]. In 1997, Lederle et al.[13] stated that diabetes was negatively associated with risk of abdominal aortic aneurysm (AAA) for the first time in the Aneurysm Detection and Management Veterans Affairs Cooperative Study. Moreover, some studies have reported that the AAA enlargement progresses more slowly in diabetes patients than in non-diabetes [22, 23].

Recently, increasing evidence has suggested the inverse relationship between diabetes and aortic diseases, including aortic aneurysm and AD. population study suggest that the overall pooled incidence rate of thoracic aortic aneurysm (TAA) and AAA was 15% lower in the type 2 diabetes cohort than non-diabetes cohort (3.85 vs 4.51 per 10 000 person-years), with an adjusted HR of 0.65 (95% CI: 0.56–0.74)[22]. In the meta-analysis of 17 large population prevalence studies, diabetes was also associated with decreased risk of AAA, with a pooled OR = 0.80 (95% CI: 0.70–0.90)[24]. Furthermore, a nationwide case-control study by the center for clinical research and evidence-based medicine at the university of Texas in Houston, indicated that diabetes was associated with decreased risk of TAA and AD [14]. Also, a very recent study revealed that diabetes patients are significantly less likely to have AD [15]. These findings suggested that diabetes may play a protective role in the development of AD. Our research showed consistent results with those studies. Although those studies are with some designing or methodological flaws, like incomplete data, relatively small samples, the underestimation of the diabetes incidence, etc [25], the results are interesting and favorable. This inverse relationship of diabetes between risks of aortic disease may question the traditional view of AD as a manifestation of atherosclerosis, especially in diabetes patients.

One thing meriting our consideration, however, is that only the inverse relationship between diabetes and risk of AD was shown and the exact mechanism for the beneficial effect by which diabetes may protect against the development of AD is still ambiguous. One of the possible mechanisms is that the biologic of aortic wall may be changed by diabetes. According to the recent studies, hyperglycemia associated with diabetes plays the role of stabilizing the collagen network through giving rise to cross-linking of collagen network in the aortic wall media. As for this cross-linking, it resists proteolysis and inhibits secretion of matrix metalloprotei-nases (MMPs) which is deemed as with regulating effect on aortic aneurysm formation and promoting effect on atherosclerotic plaque rupture [26]. Additionally, it was also revealed that diabetes could restrain plasm in that activates the matrix MMPs [27]. These effects could directly reduce aortic wall degradation and may also account for the thicker abdominal aortic wall observed in diabetes [28], as well as the potential protection of diabetes mellitus against AD. Another mechanism is that hyperglycemia is also associated with reduced adventitial neovascularization and decreased infiltration of inflammatory cells into the medial layer of the aorta. These processes could restrain the progression of AD by reduction of vascular smooth muscle cell death and extracellular matrix degradation [14]. Besides, medication taken by diabetes patients may contribute to the protective effect of aortic disease. According to previous study, metformin, one of the most wildly used anti-diabetic drugs, provides cardiovascular protection independent of its hypoglycemic effects by activating the AMP-activated protein kinase (AMPK) and reducing autophagy [29, 30]. Further exploration of the potential possibilities is needed to help understand the protective effects of these two conditions.

A key strength of this study is that it is one of the first to assess the correlation between AD risk and diabetes in a non-western case-control study with sizeable samples. Drawn from the same geography and population, both AD cases and control subjects were likely to be representative of the general population. Furthermore, effective methods were used to control biases which may influence the quality of case-control study. For controlling selection bias, all the AD cases were strictly chosen according to the inclusive criteria while control subjects were randomly selected. For decreasing confounding bias, all controls were matched by age and gender. Moreover, a 1: 2 matching design method was adopted to increase the statistical power. More importantly, our study will leave a foundation for future studies about the relationship between diabetes and risk of aortic dissection. Future research might focus on further identifying the exact mechanisms related to beneficial effect with a view to developing targeted pharmacological therapy or precaution for aortic dissection.

Nevertheless, a few limitations of our study deserve our consideration. First, as this is a retrospective case-control study, it is not appropriate for any casual association to be established between variables. Also, it was difficult to avoid residual unrecognized confounding variables influence and thus a large, multi-center, prospective study is necessary. Second, it is possible that control patients had been receiving regular medical care and thus were more likely to have diabetes diagnosed. Yet, compared with control patients, the acute aortic dissection may not have been previously cared for or diagnosed. Therefore, diabetes prevalence in aortic dissection group may be underestimated. Finally, due to the lack of research on relevant mechanisms in our part, further researches involving underlying mechanism related to the protective role of hyperglycemia in AD are required to reach a more definitive conclusion. Despite these limitations, our study may leave a foundation for future studies about the relationship between diabetes and risk of aortic diseases.

In conclusion, our findings indicated that diabetes was significantly associated with decreased risk of AD in our Chinese subjects. Further confirmatory results from other research conducted in different populations with different research designs (e.g. cohort studies) are required to establish this association; however, results from this investigation have demonstrated that diabetes may play a protective role in the development of AD and that relevant mechanisms related to the beneficial effect may provide a new insight into the causes, prevention and treatment of AD.

## Supporting Information

S1 Appendix The individual data of the case group. (XLSX)

S2 Appendix The individual data of the control group. (XLSX)
